# Genome‐wide development of intra‐ and inter‐specific transferable SSR markers and construction of a dynamic web resource for yam molecular breeding: Y2MD

**DOI:** 10.1002/tpg2.20428

**Published:** 2024-01-17

**Authors:** Moussa Diouf, Yedomon Ange Bovys Zoclanclounon, Pape Adama Mboup, Diaga Diouf, Erick Malédon, Ronan Rivallan, Hâna Chair, Komivi Dossa

**Affiliations:** ^1^ Département de Mathématiques et Informatique, Faculté des Sciences et Techniques Université Cheikh Anta Diop Dakar Senegal; ^2^ Laboratoire Campus de Biotechnologies Végétales, Département de Biologie Végétale, Faculté des Sciences et Techniques Université Cheikh Anta Diop Dakar Senegal; ^3^ Department of Crop Science and Biotechnology Jeonbuk National University Jeonju Republic of Korea; ^4^ UMR AGAP Institut CIRAD Petit Bourg France; ^5^ UMR AGAP Institut University of Montpellier, CIRAD, INRAE, Institut Agro Montpellier France

## Abstract

Microsatellite markers are widely used in population genetics and breeding. Despite the economic significance of yams in developing countries, there is a paucity of microsatellite markers, and as of now, no comprehensive microsatellite marker database exists. In this study, we conducted genome‐wide microsatellite marker development across four yam species, identified cross‐species transferable markers, and designed an easy‐to‐use web portal for the yam researchers. The screening of *Dioscorea alata*, *Dioscorea rotundata*, *Dioscorea dumetorum*, and *Dioscorea zingiberensis* genomes resulted in 318,713, 322,501, 307,040, and 253,856 microsatellites, respectively. Mono‐, di‐, and tri‐nucleotides were the most important types of repeats in the different species, and a total of 864,128 primer pairs were designed. Furthermore, we identified 1170 cross‐species transferable microsatellite markers. Among them, 17 out of 18 randomly selected were experimentally validated with good discriminatory power, regardless of the species and ploidy levels. Ultimately, we created and deployed a dynamic Yam Microsatellite Markers Database (Y2MD) available at https://y2md.ucad.sn/. Y2MD is embedded with various useful tools such as JBrowse, Blast, *insilico*PCR, and SSR Finder to facilitate the exploitation of microsatellite markers in yams. This study represents the first comprehensive microsatellite marker mining across several yam species and will contribute to advancing yam genetic research and marker‐assisted breeding. The released user‐friendly database constitutes a valuable platform for yam researchers.

## Core Ideas

- Whole genome assemblies of the four yam species were screened for the detection of microsatellites.
- A set of 1170 cross‐species transferable microsatellite markers were identified.
- An experimentally validated subset of 17 markers has shown consistent discriminatory power with respect to species and ploidy levels.
- A user‐friendly and dynamic web portal, the Yam Microsatellite Markers Database (Y2MD), has been developed and made freely available at https://y2md.ucad.sn/.
- The database includes a number of useful tools such as JBrowse, *insilico*PCR, SSRFinder, and Blast.

**Abbreviations**
AFLP: amplified fragment length polymorphism
GMATA: Microsatellites Analyzing Tool Package
GUI: graphical user interface
PCR: polymerase chain reaction
PHP: Hypertext Preprocessor
QTL: quantitative trait loci
RAPD: random amplified fragment DNA
RFLP: restriction fragment length polymorphism
SNP: single nucleotide polymorphism
SSR: simple sequence repeat
Y2MD: Yam Microsatellite Markers Database

## 1 INTRODUCTION

Microsatellites, also termed simple sequence repeats (SSRs), are tandemly repeated DNA sequences generally spanning 1–6 bp (Ellegren, 2004). SSRs are ubiquitous in the genome of higher organisms and exhibit genetic polymorphism at inter‐ and intra‐species levels (Amiteye, 2021; Bruford & Wayne, 1993; Ellegren, 2004). Therefore, they have been widely used in population genetics (Chen et al., 2022; Sapkota et al., 2022), genotyping, marker‐assisted breeding, genome‐wide association analysis (Jha et al., 2021; Kim et al., 2021; C. Li et al., 2014), forensic investigation (D. Li et al., 2021; J. Wang, Sun et al., 2022), evolutionary studies (de Freitas et al., 2022; Nowicki et al., 2021; Song et al., 2021), and conservation genetics (Dong et al., 2022; Ming et al., 2022; Rodríguez‐Peña et al., 2018).

Considering the low level of difficulty regarding the technical aspect of setting an SSR‐based genotyping core facility, the relatively inexpensive cost of reagents, and the level of throughput, SSRs markers are quite affordable and implementable in a standard laboratory (Herrera & Ghislain, 2013). Besides, SSRs can exhibit good discriminatory power, are randomly present in the genome, multi‐allelic, and can be located in the genic region, enabling the genetic dissection of traits of interest (Ellegren, 2004). Microsatellites also exhibit co‐dominance with Mendelian inheritance, facilitating their traceability within different breeding populations (Normann et al., 2018). Therefore, owing to their simple identification and high reproducibility, many researchers have relied on SSRs (X. Wang & Wang, 2016).

The development of molecular marker databases has been extensively promoted in economically important plants such as *Sesamum indicum* (Dossa et al., 2017), *Vicia faba* (Mokhtar et al., 2020), *Nicotiana* spp. (X. Wang et al., 2018), legumes species (*n* = 13) (Duan & Kaundal, 2021), horticultural species (*n* = 112), and *Anemone coronaria* (Martina et al., 2022). Since the usage of an online database is based on a graphical user interface, the database offers researchers a user‐friendly path for the rapid identification of molecular markers without a pre‐requisite expertise in command line scripting. A web database concentrates all information in one platform and assists researchers in designing markers related to a trait of interest (Dossa et al., 2017).

Yams (*Dioscorea* spp.) represent an important staple food crop, feeding over 300 million people in tropical and subtropical regions (Mignouna et al., 2008). The biggest producer country is Nigeria with 70% of the global production, followed by Ghana and Côte d'Ivoire (FAOSTAT, 2021). The genus contains ∼650 species (Virouel et al., 2018). As a cash crop, yams contribute to the income of more than 60 million people (Asiedu & Sartie, 2010), with a production of 74 million tons and an economic market value of USD 21 billion estimated in 2020 (United Nations Statistics Division, 2022). Besides, in West Africa where yam is the most cultivated in the so‐called “yam belt” (from Western Cameroon to central Côte d'Ivoire) (Sarcelli et al., 2019), more than five million people directly benefit from yam culture (Mignouna et al., 2020), making this crop a valuable resource for food security and poverty alleviation in this part of the African continent (Darkwa et al., 2020).

To accelerate yam breeding programs, attention has been paid to the development of polymorphic DNA markers. Restriction fragment length polymorphisms (RFLPs) were first developed to investigate the origin and phylogeny of cultivated Guinea yams and wild relatives by using chloroplast DNA and nuclear ribosomal DNA (Terauchi et al., 1992). Later on, amplified fragment length polymorphism (AFLP) and random amplified fragment DNA (RAPD) were employed for genetic diversity assessment (Egesi et al., 2006; Malapa et al., 2005; Zannou et al., 2009), yam mosaic virus resistance markers identification (Mignouna, Abang, et al., 2002), and genetic linkage map construction (Mignouna, Mank, et al., 2002).

Furthermore, due to their high level of polymorphism, SSRs were developed from cultivated and wild yam species (Andris et al., 2010; Hochu et al., 2006; Mignouma et al., 2003; Mizuki et al., 2005; Silva et al., 2014; Siqueira et al., 2011; Tamiru et al., 2015; Terauchi & Konuma, 1994; Tostain et al., 2006; H. Wang et al., 2022). Nonetheless, less than 100 SSR markers are available in yams to date, and no information on their genome coverage is available (Korsa et al., 2022; Loko et al., 2016; Nwogha et al., 2022; Otto et al., 2015; Tostain et al., 2007). Hence, generating more SSR markers will surely empower researchers in their routine tasks such as paternity testing, variety identification, and quantitative trait loci (QTL) detection.

Meanwhile, with the help of short and long reads, as well as chromosome conformation sequencing technologies, good‐quality genomes were recently released in yams. These data have enabled the identification of genomic regions associated with agronomically important traits, such as sex determination systems (Cormier et al., 2019; Sugihara et al., 2020; Tamiru et al., 2017), anthracnose resistance, tuber oxidative browning, and tuber starch content (Bredeson et al., 2022), as well as post‐harvest hardening (Siadjeu et al., 2021).

To date, the genomes of four species, *Dioscorea alata* (Bredeson et al., 2022), *Dioscorea rotundata* (Tamiru et al., 2017), *Dioscorea dumetorum* (Siadjeu et al., 2020), and *Dioscorea zingiberensis* (Cheng et al., 2021), are publicly available. The genomes exhibit a high level of collinearity (Bredeson et al., 2022), suggesting that SSRs might be highly transferable from one species to another, even to those wild and minor cultivated species that have not yet been sequenced. Therefore, establishing a comprehensive SSR database for yam researchers is fundamental for genetic studies.

The present study was designed to (i) mine the genome of the four yam species for the identification of single‐species and cross‐species transferable SSR markers, (ii) create primer sets for the markers, (iii) evaluate the discriminatory power of selected markers, and (iv) build a user‐friendly digital web resource to support yam genetic research.

## 2 MATERIALS AND METHODS

### 2.1 Genomic sequence resources

A total of four genome sequences were retrieved from the National Center for Biotechnology Information genome portal, including *D. alata* cultivar TDa95/00328 (GenBank assembly accession: GCA_020875875.1), *D. dumetorum* (GenBank assembly accession: GCA_902712375.1), *D. rotundata* cultivar TDr96_F1 (GenBank assembly accession: GCA_009730915.2), and *D. zingiberensis* isolate CJ2019 (GenBank assembly accession: GCA_014060945.1) (Table S1). Besides, we added a newly assembled chromosome‐scale assembly (unpublished data) of *D. alata* cultivar Kabusa. Kabusa is a widely grown variety in the West Indies.

**Core Ideas**

- Whole genome assemblies of the four yam species were screened for the detection of microsatellites.
- A set of 1170 cross‐species transferable microsatellite markers were identified.
- An experimentally validated subset of 17 markers has shown consistent discriminatory power with respect to species and ploidy levels.
- A user‐friendly and dynamic web portal, the Yam Microsatellite Markers Database (Y2MD), has been developed and made freely available at https://y2md.ucad.sn/.
- The database includes a number of useful tools such as JBrowse, *insilico*PCR, SSRFinder, and Blast.

### 2.2 Identification of microsatellites and primer design

SSRs were searched using the Genome‐wide Microsatellites Analyzing Tool Package (GMATA) (X. Wang & Wang, 2016), and primers were designed from the flanking sequences (200 bp on each side) to probe the genomic sequences. The genome assembly was imported into the GMATA “SSR identification” module in the FASTA format. The microsatellite search parameters applied for SSR detection are as follows: minimum length (nt): 1, maximum length (nt): 6, minimum number of repeats: 5.

Using custom python scripts, only sequences with a size greater than 10 bp were retained. The SSR locus information files generated (.ssr) contain the start and end positions of the SSRs on the chromosomal sequence, the SSR template, and the number of repeat units.

The genome assemblies in FASTA format and the output files (.ssr) generated by the “SSR identification” module were imported into the “Marker Designing” module of GMATA. Primer design parameters were as follows: minimum amplicon size: 100 bp, maximum amplicon size: 400 bp, optimal annealing temperature: 60°C, flanking sequence length: 200 bp, maximum model length (flanking sequence + SSR): 2000 bp. The output file (.seq) included the repeats centered on 200 adjacent sequence bases on both sides. Output files with extensions .mk and .sts were created, containing left and right primer sequences, hybridization temperatures, primer positions on chromosomes, and expected amplicon sizes.

The identified SSRs were classified into genic and non‐genic SSRs for *D. alata* and *D. rotundata* for which genome annotations are available. Subsequently, the functional enrichment of genes containing SSRs was performed with KOBAS‐i webserver (Bu et al., 2021) in order to find out their biological significance.

### 2.3 In silico polymerase chain reaction

To validate markers, generate amplicons, and assign marker positions across the *Dioscorea* genomes, the e‐Mapping module was used to run the e‐PCR algorithm (Schuler, 1997). This module was also used for intraspecific (the two *D. alata* genomes) and interspecific marker mapping at the genome scale. In e‐PCR, FASTA files containing all genome assemblies and newly developed markers created with the Marker designing module (.sts files) were imported as “Sequence File” and “Marker File,” respectively. The gap (‐n) and max. indel (‐g) parameters were set to 2 and 1, which means that two mismatches and one insertion are allowed. The output file (.emap) provides detailed marker amplification information with calculated amplification sizes and chromosomal targets and identifies single locus and multilocus markers, ultimately producing those that are potentially polymorphic (different sizes of PCR products).

### 2.4 Plant materials, DNA isolation, and PCR experiment

Leaf samples from 10 yam species (*D. alata*, *D. rotundata*, *D. dumetorum*, *Dioscorea bulbifera*, *Dioscorea trifida*, *Dioscorea transversa*, *Dioscorea esculenta*, *Dioscorea cayenensis*, *Dioscorea abyssinica*, and *Dioscorea nummularia*) including cultivated and wild species were used for DNA extraction. Six *D. alata* genotypes with different ploidy levels (14 M, 74F, Pyramide, Kabusa [2x], CT148 and CT198 [4x]) and two *D. rotundata* (Dr1 and Dr2) were included (Table S2). Plant samples were available in the germplasm collection of CIRAD, Guadeloupe. DNA was extracted based on the mixed alkyl trimethyl ammonium bromide method (Cummings & Wood, 1989). Prior to the PCR experiment, the quantity and quality of the extracted DNA were consecutively assessed on 1% agarose gel and Invitrogen Qubit Flex Fluorometer. A total of 18 SSR primers were randomly selected from different chromosomes/contigs of the four species (Table 1). In this study, we opted to analyze 18 markers for a practical reason: we aimed to use no more than three PCR plates (each containing 96 samples). These markers exhibited interspecific amplifications through the e‐PCR. M13 tail (CACGACGTTGTAAAACGAC) was added to the primers. The PCR reaction was performed using the QIAGEN kit under the conditions as follows: 95°C for 5 min, 10 cycles of 30 s at 95°C, 60°C for 1 min 30 s and 72°C for 30 s, 25 cycles of 30 s at 95°C for 1 min 30 s, and 72°C for 30 s, followed by 30 min at 60°C. The migration of the PCR products was conducted on the ABI 3500xL (Thermo Fisher Scientific Inc.). Analysis of microsatellite profiles was performed with Genemapper v6.0 (Applied Biosystems).

**TABLE 1:** Characteristics of the simple sequence repeat (SSR) primers used for genotyping and their positions on the reference genome *D. alata* TDa95.

Locus	Repeat	Chromosome	Start	End	Forward_primer (5′−3′)	Reverse_primer (5′−3′)
YMac2	(A)12	LG1	226,041	226,052	AGATGCCACGACGCAAACTT	GCAGCCGATCCTCAAAGGTC
YMar4	(TA)7	LG10	29,804,658	29,804,671	CAGGGCAAGAAGAGGGCAAG	GCTTTTCCAGTCACCTGTCCA
YMac15	(T)12	LG11	11,708,941	11,708,952	AGGCTTGGGCGAACTTTTGA	AGGGCTCGTCGTTTTGACCT
YMar11	(TTG)6	LG12	16,599,891	16,599,908	CTCTGCCAAGGCCAAGAAGG	AAACCAGCTGGCAGCATGTT
YMac33	(A)11	LG13	140,737	140,747	GCTTGCTGATAAAAACCGGAGC	TCTGGAGGAGTGTTGTCGAGA
YMar28	(TA)23	LG14	21,204,635	21,204,680	AGCTCATGGTTGGTTGCACA	GGCCATACACTTTGCCTCCC
YMac45	(AC)9	LG15	15,318,628	15,318,645	TGAGACCACTTCGTGTCTTGAG	GCACCTTCATGATGCAGCCT
YMar39	(A)10	LG17	17,125,279	17,125,288	AGACCCAAGCTGCAAAGCAA	TGACAGAGCCAACACAGAGGA
YMar44	(AT)13	LG18	24,532,026	24,532,051	TCCAGGAGCTTGGGTGATGT	GAAGACCTTCCCCACATGCA
YMac59	(ATC)7	LG19	2,365,155	2,365,175	CGCCCATTCCCTCAAAAGCT	AGCCGGGATGTTGGACAAAG
YMar65	(A)15	LG2	1,704,788	1,704,802	ACTTGCATACCCAACCCCAC	TACTGAGGAAGGTGCTGCCA
YMac82	(AT)18	LG20	13,142,390	13,142,425	GGCTTCATTGGCTCTGGCTT	GCCAGCAAGAAATGGACCCA
YMar79	(CCA)9	LG3	815,571	815,597	ACTATGTCAGCACCCACCCC	AAACTGGGGAGCAGTTGCAC
YMac92	(A)11	LG4	5,139,570	5,139,580	GCAGTTGACACACACGAGACA	CATGTGGATCGCTCATGCCA
YMar90	(GA)9	LG5	13,506,295	13,506,312	AGAGTGCCAGCCTCAGTTACA	AGGAAGGCGCTCTTTGATGC
YMac118	(A)10	LG6	12,894,570	12,894,579	TCATCCTCTCCCTGTCAGCG	GTAGTTGCTGCCATGGTGCA
YMar128	(GA)15	LG7	3,819,341	3,819,370	CGGCGACAAGAGAACAAGCA	CCACCTCCTTGAACATCGCC
YMac135	(TCA)13	LG9	16,767,472	16,767,510	TGCAGAAGTTCACCGCTCAG	CCTTCCTTGCTCAGCTGTGG

### 2.5 SSR genotyping data analysis

The genotyping data served to perform a hierarchical clustering to depict the genetic relationships between the 16 accessions (Table S2). The clustering was conducted in R v4.2.2 (R Core Team, 2022) following the unweighted pair group method with the arithmetic mean method using the function *hclust()* of the package base. The resulting tree was rendered with the function *fviz_dend()* of the package factoextra v1.0.7 (Kassambara & Mundt, 2020). For comparative analysis purposes, a principal component analysis was also executed with the function *PCA()* of the package FactoMineR (Husson et al., 2020). The eigenvalues following each principal component were rendered with the function *fviz_screeplot()* of the package factoextra v1.0.7 (Kassambara & Mundt, 2020). The species projections in the factorial plan (1, 2), (1, 3), and (1, 4) were rendered with the function *fviz_pca_ind()* of the package factoextra v1.0.7 (Kassambara & Mundt, 2020).

### 2.6 Construction and deployment of Yam Microsatellite Markers Database

**FIGURE 1:**
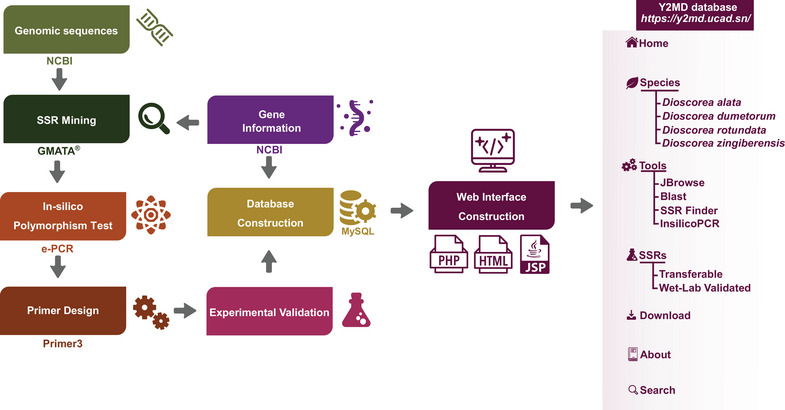
A diagram depicting the structure of Yam Microsatellite Markers Database (Y2MD). NCBI, National Center for Biotechnology Information; GMATA, Genome‐wide Microsatellites Analyzing Tool Package.

The Yam Microsatellite Markers Database (Y2MD) conception relied on three major steps detailed in Figure 1: (i) data compilation in SQL database, (ii) back‐ and front‐end web construction, and (iii) tools integration.

In brief, using the generated SSR markers datasets, the Y2MD SQL database was built. Thus, the Y2MD server was implemented using Linux, Apache, MySQL, and the Hypertext Preprocessor (PHP) web application platform. The user‐friendly web interface was then developed with the aid of JavaScript and the PHP. To enable the users to conduct their own analyses conveniently, additional functionalities such as JBrowse (Skinner et al., 2009), Blast (Altschul et al., 1990), SSR Finder (http://www.biophp.org/minitools/microsatellite_repeats_finder/), and *insilico*PCR (http://www.biophp.org/minitools/pcr_amplification/) were embedded. The website is freely accessible at https://y2md.ucad.sn/.

## 3 RESULTS

### 3.1 Detection, distribution, and characteristics of microsatellites detected on yam genomes

**FIGURE 2:**
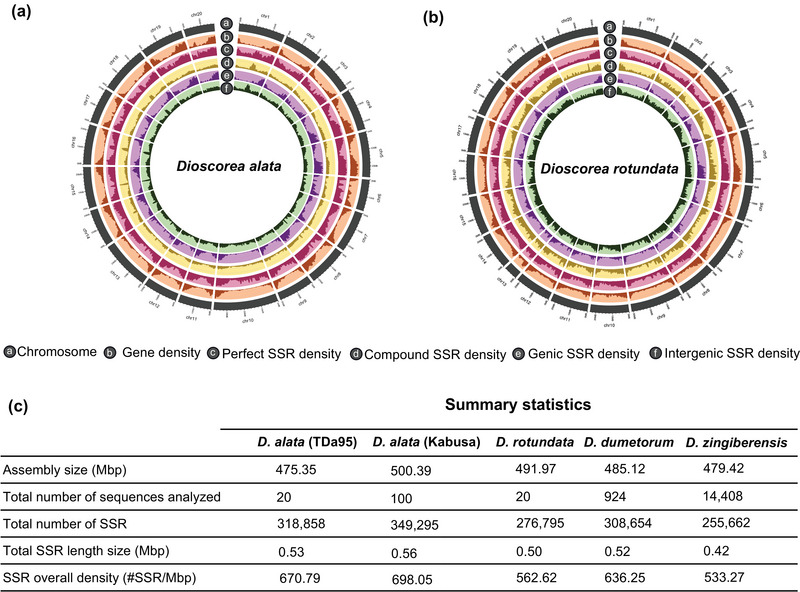
Distribution and summary statistics of simple sequence repeats (SSRs) identified in yam genomes. Circos plots depicting the SSR density within the chromosome‐scale genomes of *Dioscorea alata* (TDa95) (a) and *D. rotundata* (b). Summary statistics of SSR counts in the five studied genomes (c).

The genome sequences of the four yam species were utilized for the identification of SSRs. In total, 318,713, 322,501, 307,040, and 253,856 SSRs were detected in *D. alata*, *D. rotundata*, *D. dumetorum*, and *D. zingiberensis* genomes, respectively, with corresponding densities of 662.86, 552.22, 633.07, and 529.97 SSR/Mbp (Figure 2; Figure S1). At the intraspecific level, the genome of the *D. alata* cultivar Kabusa contained 346,986 SSRs with a density of 693.972 SSR/Mbp, surpassing that of *D. alata* cultivar TDa95/00328.

**FIGURE 3:**
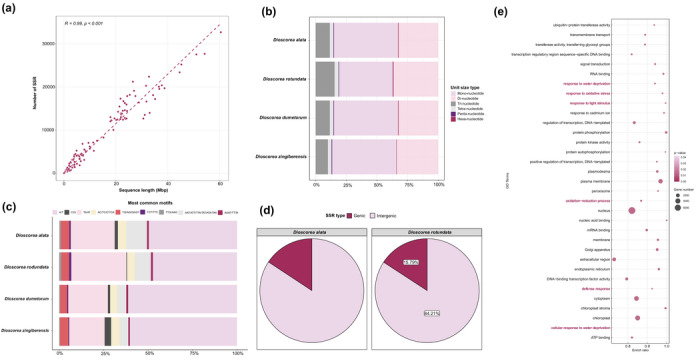
Correlation between chromosome length and number of simple sequence repeats (SSRs) (a); proportions of the nucleotide repeats type (b); distribution of the most common patterns in the four species (c); proportions of genic and non‐genic SSRs in *D. alata* and *D. rotundata* (d); bubble plot showing significantly enriched gene ontology terms related to genic SSRs regions. The gene ontology terms highlighted in red are those that are agronomically relevant (e).

The number of SSRs per chromosome is strongly correlated with the chromosome lengths (Figure 3a). A total of 658 different motifs were observed in *D. alata*, 843 in *D. dumetorum*, 762 in *D. rotundata*, and 753 in *D. zingiberensis* (Figure 3b). Mono‐, di‐, and tri‐nucleotides are the most important types of repeats in the different species (Figure 3c).

The obtained motifs are notably rich in A/T; A and T mononucleotides are the most represented, constituting an average of 55% of the detected SSRs. Additionally, 20% of the SSRs are composed of AT and TA dinucleotides. For tri‐nucleotides, repeats (AAT/ATA/ATT/TAA/TTA) account for 7.5%. Hexa‐ and penta‐nucleotides exhibit relatively low frequencies. Approximately 84% of the SSRs are localized in the intergenic regions of *D. alata* and *D. rotundata* genomes (Figure 3d). This analysis was not performed for other species due to the lack of proper genome annotation. Genes containing SSRs were analyzed for functional enrichment and showed enrichment in a wide array of biological attributes. Notably, several agronomically relevant gene ontology terms were identified, including response to water deprivation (GO:0009414), response to oxidative stress (GO:0006979), response to light stimulus (GO:0009416), defense response (GO:0006952), and inflorescence development (GO:0010229) (Figure 3e; Table S3).

### 3.2 Development of genome‐wide SSR primers

A total of 864,128 unique primer pairs were generated from the 1,201,570 SSRs extracted from the four *Dioscorea* genomes: *D. alata* (221,606), *D. rotundata* (233,593), *D. dumetorum* (208,306), and *D. zingiberensis* (200,623). The distribution of primers across the genomes is uneven. The identified primers and their characteristics are detailed in Table S4. Each marker was assigned an identifier based on the respective *Dioscorea* species: YMa for *D. alata*, YMr for *D. rotundata*, YMd for *D. dumetorum*, and YMz for *D. zingiberensis*, followed by consecutive numbers representing the primer positions in the genomes.

### 3.3 Cross‐species transferability of SSRs

**FIGURE 4:**
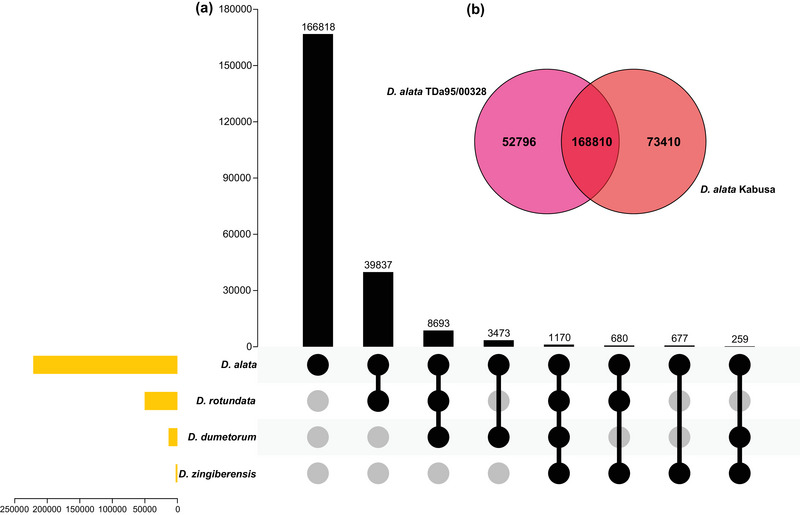
Cross‐species and transferability of simple sequence repeats (SSRs) in yams. Upset plot showing the transferable markers between four yam species (*D. alata* as the reference genome, *D. rotundata*, *D. dumetorum*, and *D. zingiberensis*) (a); Venn diagram showing the number of common and specific SSR markers detected in two *D. alata* cultivars (TDa95/00328 and Kabusa) (b).

First, we conducted a pairwise comparison of the microsatellite markers among the four yam species (Table S5). *Dioscorea alata* and *D. rotundata* displayed the highest conservation rate (54.78%), while *D. rotundata* and *D. zingiberensis* had the lowest conservation rate (1.64%). Next, to assess the cross‐species transferability of the microsatellite markers, we used *D. alata* as a reference and performed e‐PCR on the other three species. Out of 221,606 SSR markers from *D. alata* (TDa95/00328), 166,817 SSRs were specific to *D. alata*, while 39,837 SSRs could amplify *D. rotundata*, 3473 on *D. dumetorum*, and 677 on *D. zingiberensis* (Figure 4a). Overall, we identified 1170 SSR markers capable of amplifying all four species and potentially more yam species.

At the intraspecific level, we compared the SSR marker data identified in the genomes of two *D. alata* cultivars (TDa95/00328 and Kabusa). The results showed that both cultivars shared 77% of the total SSR markers (Figure 4b).

### 3.4 Validation of selected SSRs and genotyping analysis

First, to check whether the previously developed SSR markers could be found within our dataset, we extracted a total of 92 SSR markers routinely used for genotyping *D. alata* and *D. rotundata* from the literature (Korsa et al., 2022; Loko et al., 2016; Nwogha et al., 2022; Otto et al., 2015; Tostain et al., 2007; Zawedee et al., 2014). This analysis was performed using the e‐PCR method. Out of the 92 markers, 84 (91%) SSRs yielded PCR products on their respective reference genomes.

Next, to further validate our developed SSR markers, 18 markers were randomly selected from the 1170 cross‐species transferable SSRs (Figure 4a). These markers are located on different chromosomes/contigs of the four species and were utilized for wet‐lab PCR genotyping in 10 yam species (Table S2; Table 1). It is noteworthy to mention that we specifically targeted 18 SSRs for a practical reason: we aimed to use no more than three PCR plates (each containing 96 samples). Out of the 18 selected markers, 17 produced amplicons, with the exception of the marker YMar128. Sixteen markers produced amplicons in at least six species out of the 10 (Table 2). Interestingly, the marker amplifications in diploid and tetraploid *D. alata* cultivars yielded a maximum of two or four alleles, respectively. For instance, the marker YMar79 was able to clearly delineate diploid from tetraploid forms of *D. alata* (Table 2).

**TABLE 2:** Allele scoring for 18 selected simple sequence repeat (SSR) markers in 10 yam species.

Marker	*D. alata* (14 M)	*D. alata* (74F)	*D. alata* (CT148)	*D. alata* (CT198)	*D. alata* (Pyramide)	*D. alata* (Kabusa)	*D. bulbifera*	*D. transversa*	*D. rotoundata Dr1*	*D. rotoundata Dr2*	*D. trifida*	*D. dumetorum*	*D. esculenta*	*D. cayenensis*	*D. abyssinica*	*D. nummularia*
YMar02	198	199	197 + 199	197 + 199	198 + 199	196 + 197	212 + 213	198 + 199	198 + 199	198	215	209	204	198 + 199	197 + 200	196 + 199 +204
YMar04	313 + 325	325	309 + 313	309 + 313	313 + 325	313 + 327	333 + 335	313 + 315	322	322	343	349	322	320 + 322	322	311
YMar11	303	303	303	303	303	303	304	303	317	311 +317	296	304	308 +311 +317	303	314	302
YMar118	196	202	202	202	196	202	194	202	198	196	214	NA	NA	197	197	198
YMar128	NA	NA	NA	NA	NA	NA	NA	NA	NA	NA	NA	NA	NA	NA	NA	NA
YMar135	160	160	157 + 160 + 176	157 + 160 + 176	160	176	163 + 169	163 + 166 + 172	160	160	176	160 + 163	NA	157 + 160	163	163 + 166 + 172
YMar15	158	158	158 + 159	158 + 159	158	158	NA	155 + 158	157	157	155	152	155	153 + 157	156 + 157	155 + 158
YMar28	234 + 254	232 + 248	251 + 260	251 + 260	226 + 230	224	234	224	234 + 248	230 + 232	224	NA	NA	236 + 260	232 + 234 + 238 + 240	224
YMar33	181 + 183	183 + 184	181 + 184	181 + 184	183	184	170 + 189	176 + 184	184	184	NA	186	184 + 185	184	182	182
YMar39	180	180	180 + 181	181	180	180 + 182	170	154 + 157 + 181	173 + 176	176	166	171	185	173 + 176 + 1778 + 186	173	154 + 157
YMar44	192 + 206	NA	208 + 212	196 + 208	192 + 204	196 + 206	NA	184 + 192 + 196 + 200	190 + 196	190 + 196	NA	NA	NA	190 + 194 + 198 + 208	192	192 + 196
YMar45	277 + 281	277	281	281	277 + 281	279 + 281	NA	275 + 277 + 281	273	273	NA	281	259	267 + 275	275	275
YMar59	298	298	NA	NA	298	298	310	298 + 301	307 + 310	310 + 319	NA	NA	NA	310	307	301
YMar65	274 + 275	NA	274 + 277	274 + 277	275	273 + 275	NA	274	NA	NA	NA	NA	NA	NA	NA	NA
YMar79	217	208 + 217	207 + 211+ 223	207 + 211+ 220 + 223	217 + 223	220	198	207 + 211+ 221 + 225	215 + 220	214	223	236 + 242	223	208 + 214	212 + 215	221 + 225+ 229 + 236
YMar82	238 + 254	238 + 254	226 + 238 + 256	226 + 256	252 + 254	228 + 234	NA	230 + 238 + 246 + 252	238	238	NA	NA	NA	238 + 248 + 254	232 + 242 + 246 + 264	236 + 246 + 252
YMar90	294	292	292	292	292 + 304	292 + 294	332	294 + 310	306	296 + 298	332 + 340	NA	NA	296 + 314	310	294 + 310
YMar92	215	215	NA	NA	214	215 + 219	209	211 + 214	212 + 215	211 + 212	NA	215 + 223	219	211 + 212	216	211

Abbreviation: NA, not available.

Interestingly, four alleles were amplified for *D. transversa* (markers YMar44, YMar79, and YMac82), *D. cayenensis* (markers YMar39 and YMar44), *D. abyssinica* (markers YMar28 and YMac82), and *D. nummularia* (marker YMar79), indicating that the cultivars of these species used in this study are polyploids (4x or more). Moreover, a maximum of two amplified alleles were found for both cultivars of *D. rotundata*, suggesting that they are diploid types.

**FIGURE 5:**
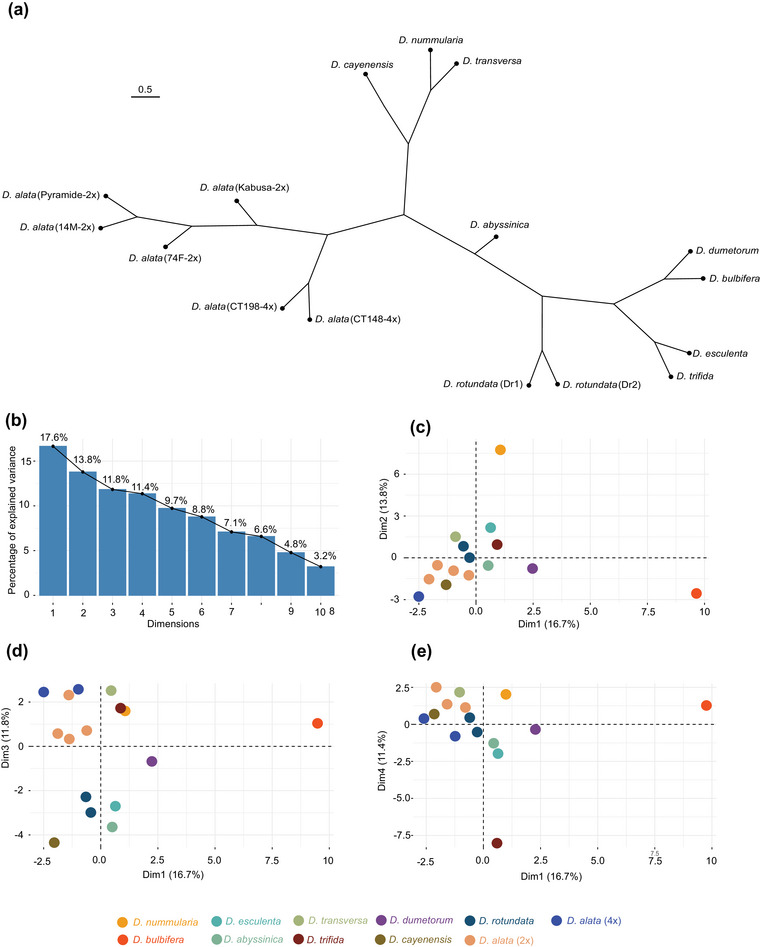
Hierarchical clustering tree depicting the relationships between 10 yam species using 17 simple sequence repeat (SSR) markers (a). The tree was constructed based on the Unweighted pair group method with arithmetic mean method. Taxa labels were arranged as follows: scientific name, genotype name, and ploidy. Ploidy information was not mentioned for the species with undetermined ploidy. The scale bar represents the length of the tree branch. Principal component analysis depicting the variability retained on each principal component (b), the projection of each species following the factorial plans (1, 2) (c) (1, 3) (d), and (1, 4) (e).

Based on the genotyping data, we conducted a hierarchical clustering analysis of the accessions and generated a tree. The tree highlighted a branch exclusively harboring *D. alata* cultivars (Figure 5).

Among *D. alata* cultivars, a clear distinction between diploid and tetraploid types was also noted, implying the robustness of the selected markers for discriminating yam accessions based on both species and ploidy criteria. The species *D. cayenensis* and *D. abyssinica* were singularly separated from the other species, while *D. transversa* and *D. nummularia* grouped together. Interestingly, the two cultivars of *D. rotundata* grouped together as expected. Meanwhile, two other groups were also identified: one consisting of *D. trifida* and *D. esculenta* and another group formed by *D. dumetorum* and *D. bulbifera*.

Interestingly, four alleles were amplified for *D. transversa* (markers YMar44, YMar79, and YMac82), *D. cayenensis* (markers YMar39 and YMar44), *D. abyssinica* (markers YMar28 and YMac82), and *D. nummularia* (marker YMar79), indicating that the cultivars of these species used in this study are polyploids (4x or more). Besides, a maximum of two amplified alleles were found for both cultivars of *D. rotundata*, suggesting that they are diploid types.

Based on the genotyping data, we performed a hierarchical clustering analysis of the accessions and generated a tree. The tree highlighted a branch exclusively harboring *D. alata* cultivars (Figure 5). Among *D. alata* cultivars, a clear distinction between diploid and tetraploid types was also noted, implying the robustness of the selected markers for discriminating yam accessions based on both species and ploidy criteria. The species *D. cayenensis* and *D. abyssinica* were singularly separated from the other species, while *D. transversa* and *D. nummularia* gathered together. Interestingly, the two cultivars of *D. rotundata* grouped together as expected. Meanwhile, two other groups were also identified: one consisting of *D. trifida* and *D. esculenta* and another group formed by *D. dumetorum* and *D. bulbifera*.

Meanwhile, from the principal component analysis (Figure 5b–e), a total of 53.7% of the total variance was retained in the top four factorial dimensions (Figure 5b). Based on the contribution of each species to the construction of factorial plans (1,2) (Figure 5c), plans (1, 3) (Figure 5d), and plans (1, 4) (Figure 5e), the species *D. nummularia* (Figure 5c), *D. bulbifera* (Figure 5c,d), and *D. trifida* (Figure 5e) clearly separated from their congeners. Moreover, a clear delineation of diploid and tetraploid *D. alata* species was also noticeable (Figure 5d), as observed in the clustering analysis.

### 3.5 Development of Yam Microsatellite Markers Database

We established a comprehensive database by integrating SSR markers and whole‐genome assemblies and annotation datasets (Figure 1). The Y2MD page contains data for four *Dioscorea* species and over 864,128 primer pairs. Additionally, all SSR data are downloadable in Excel, PDF, text, and CSV formats. The graphical user interface (GUI) menu has six tabs: “Home,” “Species,” “Tools,” “SSRs,” “Download,” and “About.”

The “Species” tab displays information on each species with the option to choose the SSR of choice based on the type of patterns, the number of repeats, the position, and so on. Information on experimentally validated SSRs and cross‐species transferable SSRs is available in the “SSRs” tab. On the “Download” page, links to the different genomes are provided.

**FIGURE 6:**
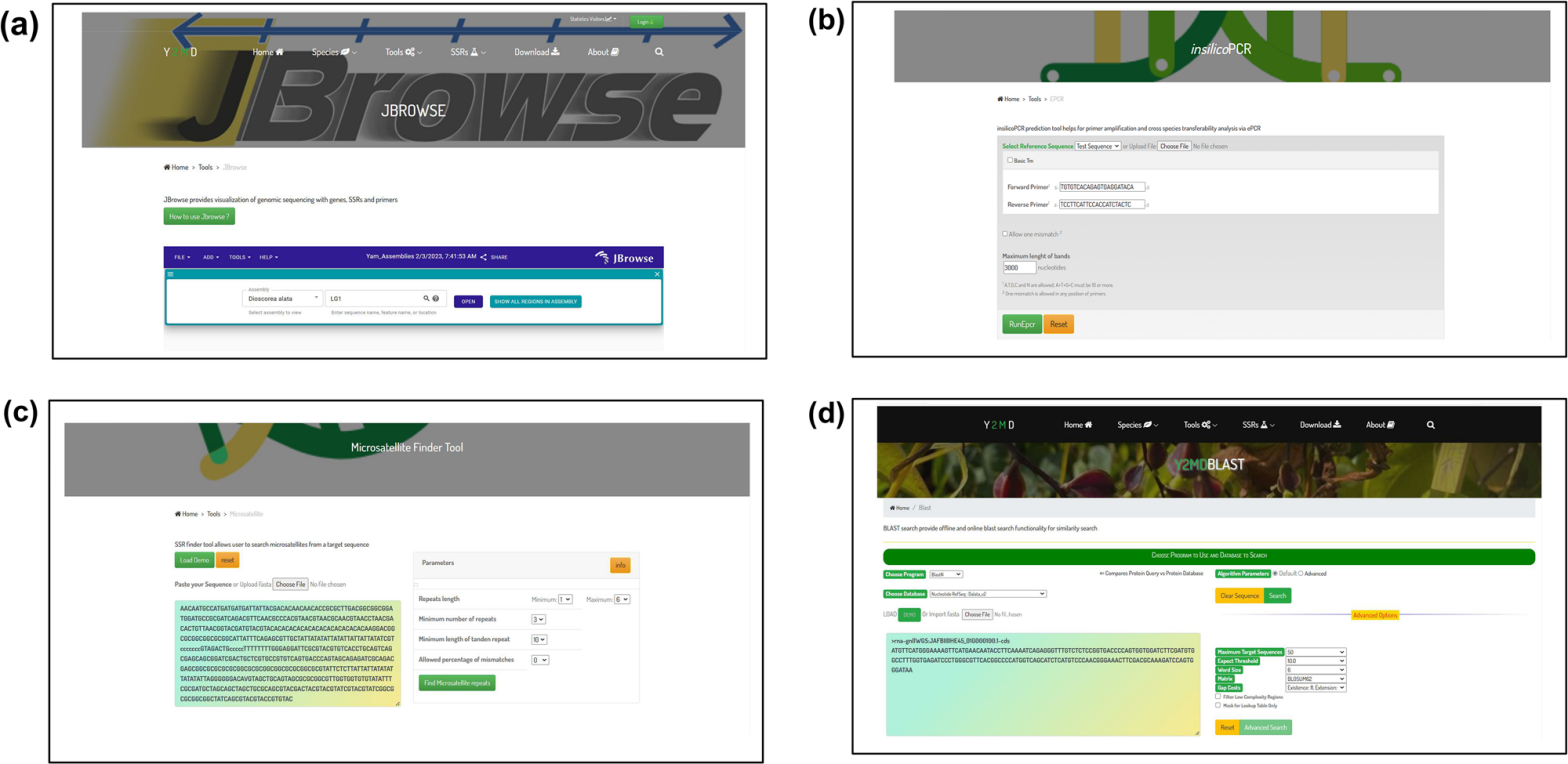
The Yam Microsatellite Markers Database (Y2MD). The website is freely available at https://y2md.ucad.sn/. The web interface of Y2MD showcasing JBrowse (a), *insilico*PCR (b), SSR Finder (c), and Blast (d) functionalities

To enable database users to perform fundamental analyses, Y2MD incorporates several functionalities, including JBrowse (Figure 6a), *insilico*PCR (Figure 6b), SSR Finder (Figure 6c), and Y2MD Blast (Figure 6d). Therefore, Y2MD empowers users to find an SSR with the SSR Finder, conduct a quick amplification check with *insilico*PCR, and blast their own sequence of interest onto a genome using the Y2MD Blast tool. With JBrowse, users can explore the genome annotation map to identify potential genes related to a targeted SSR. Overall, Y2MD offers a dynamic platform for yam researchers to remotely utilize available genomic resources in a user‐friendly GUI. The website is freely accessible at https://y2md.ucad.sn/.

## 4 DISCUSSION

### 4.1 Developing genome‐wide SSR markers in Dioscoreaceae

Microsatellite markers have proven to be effective in characterizing yam germplasms worldwide (Arnau et al., 2017; Cao et al., 2021; Obidiegwu, Asiedu, et al., 2009; Obidiegwu, Kolesnikova‐Allen, et al., 2009; Siqueira et al., 2011; Tamiru et al., 2015; Tostain et al., 2006; H. Wang et al., ^2022^). The present study enabled the extraction of a significant number of microsatellites in different yam genomes. The extracted SSRs were more concentrated in the telomeres, as reported by Jian et al. (2021). *Dioscorea zingiberensis* (253,856 microsatellites) had fewer microsatellites compared to other yam species. Nevertheless, we observed a high correlation (*R* ^2^ = 0.99) between the SSR numbers and chromosome lengths. Our findings are similar to the report of Dossa et al. (2017) in sesame, where the relationship between chromosome length and the number of SSRs on each chromosome showed a strong correlation (*R* ^2^ = 0.94).

Overall, the SSR densities observed in yam species (ranging from 529 to 662 SSR/Mbp) are similar to those of sesame (507 SSR/Mbp) but lower than other species such as Arabidopsis (*Arabidopsis thaliana*) and rice (*Oryza sativa*) (875 and 807 SSRs/Mbp, respectively) (Dossa et al., 2017; Lawson et al, 2006). Comparing two genome assemblies of *D. alata* (Kabusa and TDa95/00328) highlighted major differences in terms of SSR numbers and densities. We speculate that the completeness of the genome assemblies and the genetic distances (data unpublished) between the two accessions may explain these differences.

Among the observed motifs, mononucleotides, which account for 50% of SSRs, are the most represented. However, they are not ideal targets for PCR markers (Clarke, 2001). The most abundant types of SSRs are mono (A/T)n, di (AT/TA)n, poly (ATA/ATT/TAT/TAA)n, and therefore rich in AT. Similar results were obtained in potato (*Solanum tuberosum*) (Jian et al., 2021), Arabidopsis (Cavagnaro et al., 2010), sesame (Uncu et al., 2015), sorghum (*Sorghum bicolor*) (Yonemaru et al., 2009), and rice (McCouch et al., 2002).

A cost comparison of SSR and SNP genotyping systems in yam is difficult to make, mainly because no SNP array has yet been released for the yam community. Based on our previous genotyping experience, we spent €325 as a full‐cost package for genotyping 16 yam samples with 18 SSRs (∼€20 per sample). In comparison, we spent €2150 to genotype 120 yam samples with 77 KASPar SNPs (around €17 per sample). Although the cost of SSR genotyping is slightly higher than SNP markers in our case, only 72% of the KASPar SNP genotyping data were usable, compared to 94% for SSR markers. Larsen et al. (2018) revealed that an accurate cost comparison between SSR marker and SNP marker systems is quite impossible. They showed that the costs of generating SNP data were 10 times higher than the laboratory costs of obtaining the SSR data in apple. But they specified that generating SSR data was much more labor‐intensive than SNP genotyping since it requires many manual steps. Bernard et al. (2019) showed that the use of only 13 SSR markers was sufficient to cover the genetic structure in the walnut germplasm collection, while hundreds of SNPs were required to achieve the same level of resolution. Similarly, Choudhury et al. (2023) demonstrated that 30 unlinked SSR markers were more powerful than 32,782 SNPs for diversity analysis of Indian rice. In addition to these advantages, the SSR marker system can be fully integrated into laboratories. Nonetheless, SNP genotyping is still a better option when working on large yam germplasms.

### 4.2 Cross‐species transferability of the SSR markers

The identification of polymorphic SSR markers will support research on yam genetics and breeding. At the genome scale, we identified 1170 cross‐species transferable markers in the studied four *Dioscorea* species. Previously, Tamiru et al. (2015) showed that 94.4% and 56.7% of SSR markers from *D. cayenensis* could be successfully transferred to *D. rotundata* and *D. alata*, respectively. Likewise, Tostain et al. (2006) developed 16 polymorphic SSR markers transferable to *D. alata*, *D. abyssinica*, and *D. praehensilis*. Transferring markers between species is often considered a better investment than developing and testing new markers, especially when available funding is limited (Fan et al., 2013). Because, in most breeding programs, different yam species are grown and evaluated together, having SSR marker sets applicable to different species is highly useful. We also noticed that the number of cross‐species transferable markers decreased significantly when *D. zingiberensis* was included. Indeed, Bredeson et al. (2022) compared the *D. alata* reference genome with the genomes of *D. rotundata* and *D. zingiberensis* and revealed substantial conservation of chromosome structures between *D. alata* and *D. rotundata*, but to a lesser extent with *D. zingiberensis*.

When several genome assemblies are available within the same species, in silico detection of polymorphic markers is resource‐efficient in terms of time and cost. This approach has been successfully applied in sesame, spinach, and *Perca fluviatilis* (Bhattarai et al., 2021; Dossa et al., 2017; Xu et al., 2022). We compared the common SSR markers from the genomes of *D. alata* (TDa95/00328 and Kabusa) and obtained 93.92% polymorphic SSR markers. In other species, such as potato, tomato, and bell pepper, the levels of intraspecific polymorphisms appear to be rather limited (Stàgel et al., 2008); therefore, more efforts were needed to develop informative SSR markers in these species. In *D. alata*, the high SSR polymorphic rates could be attributed to the dioecy nature of the species.

### 4.3 Validating selected SSRs

In *Dioscoreaceae*, only a limited number of SSR markers have undergone experimental (wet‐lab) validation (Andris et al., 2010; Arnau et al., 2017; Cao et al., 2021; Malédon et al., 2023; Obidiegwu, Asiedu, et al., 2009; Obidiegwu, Kolesnikova‐Allen, et al., 2009; Siqueira et al., 2011; Tamiru et al., 2015; Tostain et al., 2006; H. Wang et al., ^2022^), most of which originate from *D. alata* and *D. rotundata*. In contrast, other species such as rice (McCouch et al., 2002), sesame (Dossa et al., 2017), and cowpea (*Vigna unguiculata*) (Jasrotia et al., 2019) have a large number of markers that have been identified, experimentally characterized, and made available for genetic studies and breeding.

In this study, we extracted 92 markers from the literature (markers tested through wet‐lab experiments), and most of them were found among the markers identified in our study. The e‐PCR parameters, which allow only two mismatches and one insertion for the primers, could be partly responsible for the absence of PCR products for the wet‐lab tested markers that are missing. This analysis also provided the exact locations of these SSRs in the yam genomes, enabling researchers to select markers from different chromosomes for enhanced discriminatory power.

Out of the 18 selected SSR markers from this study, 17 were able to PCR amplify genotypes of several yam species. The experimentally tested markers were from different chromosomes and demonstrated robust discriminatory power by clearly delineating the studied yam species and ploidy levels. In yam species, the basic chromosome number is 20, and most species harbor different cytotypes (Abraham et al., 2013; Arnau et al., 2009; Bousalem et al., 2010; Scarcelli et al., 2005; Sughihara et al., 2021). Prior to our work, *D. rotundata* cultivars were reported to be mainly diploid using flow cytometry, isoenzymes, DArTseq SNP, and a set of six microsatellite markers (Gatarira et al., 2021; Scarcelli et al., 2005). Both *D. rotundata* cultivars investigated in this study were found to be diploid. Additionally, the tested SSR markers also showed promising accuracy even at the intra‐species level with a clear classification of diploid and tetraploid forms of *D. alata* cultivars. Based on the allele numbers, the yam species, including *D. transversa*, *D. abyssinica*, *D. cayenensis*, and *D. nummularia*, have cultivars with tetra‐ or higher ploidy levels (Gatarira et al., 2021; Sughihara et al., 2021). Therefore, we provided in the present study SSR markers that can be used at low cost for ploidy checks and species differentiation in yams.

### 4.4 Deployment of Yam Microsatellite Markers Database for yam breeders

Several databases containing SSR marker data from various species are available to the public. However, there is currently no database dedicated to the study of yam microsatellites. Establishing a yam microsatellite marker database would serve as a valuable platform for advancing genetic evaluation, genomic research, and yam breeding. While the Pan Species Microsatellite Database (Du et al., 2020) includes SSR data from 18,408 organisms, its utility is limited due to the absence of features such as ePCR, JBrowse (Buels et al., 2016), and Blast (Altschul et al., 1990). This limitation is also observed in the Tea Microsatellite Database (Dubey et al., 2020) and SSRome (Mokhtar & Atia, 2019).

In this study, we have compiled all generated microsatellite data into the Y2MD. Y2MD incorporates various useful tools, including JBrowse, Blast, SSR Finder, and *insilico*PCR. These tools are designed to facilitate the localization, SSR detection, and polymorphism analysis of both existing and new experimental yam SSR markers/nucleotide sequences. Y2MD has been developed as a highly user‐friendly database, enabling all users, especially those with limited knowledge or resources in bioinformatics, to analyze their SSR‐related data.

## 5 CONCLUSION

In this study, a graphical interface containing the SSR markers of four commercially important *Dioscorea* species was constructed and made available to the public via https://y2md.ucad.sn/. To perform the database construction, microsatellites from four *Dioscorea* species were first extracted, and primers were designed based on the flanking sequences of these microsatellites. In total, 1,201,570 microsatellites were extracted from all species, resulting in 864,128 designed primer pairs. Subsequently, additional analyses were conducted, including cross‐species transferability and determination of potential polymorphic markers, which are determining factors in plant genotyping. Experimental validation of selected markers was also performed to lend credence to our datasets. Overall, the SSRs developed in this study will be useful for the genetic characterization of yam germplasms. All these data have been compiled into Y2MD and will be of great use to the yam community.

With the rapid development in genome sequencing projects, we anticipate more yam genomes to become available. Consequently, we plan to expand Y2MD to include new *Dioscorea* species as soon as their genome sequences are accessible. Additionally, Y2MD will be enhanced by incorporating new tools and functionalities, such as the integration of Primer3, introduction of molecular markers associated with QTLs, and inclusion of new wet‐lab validated SSRs. We also intend to integrate SNP marker data into Y2MD, evolving it into a new platform with various multi‐omics data, such as transcriptome and metabolome data.

## AUTHOR CONTRIBUTIONS

**Moussa Diouf**: Data curation; formal analysis; visualization; writing—original draft. **Yedomon Ange Bovys Zoclanclounon**: Data curation; formal analysis; visualization; writing—original draft. **Pape Adama Mboup**: Data curation; formal analysis. **Diaga Diouf**: Conceptualization; data curation; formal analysis; writing—review and editing. **Erick Malédon**: Formal analysis; validation. **Ronan Rivallan**: Formal analysis; validation. **Hâna Chair**: Formal analysis; validation; writing—review and editing. **Komivi Dossa**: Conceptualization; data curation; formal analysis; funding acquisition; methodology; project administration; resources; supervision; validation; writing—review and editing.

## CONFLICT OF INTEREST STATEMENT

The authors declare no conflicts of interest.

## Supporting information

Supplemental Table S1. List of sequences retrieved from NCBI.
Supplemental Table S2. List of yam accessions used for SSR genotyping along with their ploidy level and origin.
Supplemental Table S3: Gene ontology analysis of identified genic SSRs.
Supplemental Table S4. Statistics of SSRs detected for all species investigated in this study.
Supplemental Table S5. Pairwise comparisons for SSR conservation among yam species.

Supplemental Figure 1: Overview of the high‐density SSR physical map in *Dioscorea alata* (a) and *Dioscorea rotundata* (b). The bar represents the number of SSR markers within a 1‐Mb window.

## Data Availability

All SSR data generated and the genomes used are available online at https://y2md.ucad.sn/. The genome sequence of *Dioscorea alata* cultivar Kabusa has not been made available publicly yet, but it can be shared upon request to the corresponding author.
