# Supplementary material for: Genome‐wide development of intra‐ and inter‐specific transferable SSR markers and construction of a dynamic web resource for yam molecular breeding: Y2MD
Source: Plant Genome. 2024 Jan 17;17(2):e20428. doi: 10.1002/tpg2.20428 (PMC12807095; doi:10.1002/tpg2.20428)
Supplement: Supplementary file 1 [file TPG2-17-e20428-s002.docx]

**Supplemental Figure 1**: Overview of the high-density SSR physical map in *Dioscorea alata (*a) and *Dioscorea rotundata (b)*. The bar represents the number of SSR markers within a 1-Mb window.
